# Hémocholécyste compliquée d’une rupture de la vésicule biliaire

**DOI:** 10.11604/pamj.2019.34.45.18682

**Published:** 2019-09-24

**Authors:** Haithem Rejab, Aymen Trigui, Hazem Ben Ameur, Youssef Majdoub, Rahma Daoud, Amira Akrout, Salah Boujelbene, Rafik Mzali

**Affiliations:** 1 Faculté de Médecine de Sfax, Service de Chirurgie Viscérale et Générale, Hôpital Habib Bourguiba, Sfax, Tunisie

**Keywords:** Hémocholécyste, hémopéritoine, anticoagulant, Hemocholecyst, hemoperitoneum, anticoagulant

## Abstract

L'hémocholécyste est défini par la présence d'une hémorragie au sein de la vésicule biliaire. C'est une complication rare des traitements anticoagulants, elle peut évoluer vers la rupture spontanée de la vésicule biliaire se traduisant par un état de choc hémorragique. Nous rapportons le cas d'un homme de 75 ans, hypertendu, dyslipidémique et porteur d'une cardiopathie hypertensive, qui a été hospitalisé initialement dans un tableau d'hémiplégie gauche. Le patient a été alors mis sous traitement antiagrégant plaquettaire et une anticoagulation par héparine de bas poids moléculaire (HBPM) à dose préventive. Compliqué au 5^ème^ jour de traitement d'un hémocholécyste et hémopéritoine confirmé par un angio-tomodensitométrie abdominale faite en urgence. Le geste a consisté en une cholécystectomie, une hémostase du lit vésiculaire et une évacuation de l'hémopéritoine.

## Abstract (en)

Hemocholecyst is defined as a hemorrhage into the gallbladder. It is a rare complication of anticoagulant therapies which can progress to spontaneous rupture of the gallbladder with hemorrhagic shock. We report the case of a 75-year old hypertensive, dyslipidemic man with hypertensive heart disease initially hospitalized for left hemiplegia. The patient received antiplatelet and anticoagulant therapy with low molecular weight heparin (LMWH) as prevention strategy. After 5 days of treatment the patient developed hemocholecyst and hemoperitoneum, confirmed by angio-abdominal computerized tomography scan in emergency assessment. The patient underwent cholecystectomy, hemostasis of the gallbladder fossa and evacuation of the hemoperitoneum.

## Introduction

L'hémocholécyste est défini par la présence d'une hémorragie au sein de la vésicule biliaire. C'est une complication rare des traitements anticoagulants, elle peut évoluer vers la rupture spontanée de la vésicule biliaire se traduisant par un état de choc hémorragique. Avec l'imagerie en coupe, le diagnostic se fait précocement permettant ainsi une prise en charge chirurgicale rapide.

## Patient et observation

Nous rapportons le cas d'un homme de 75 ans, hypertendu, dyslipidémique et porteur d'une cardiopathie hypertensive, qui a été hospitalisé initialement dans un tableau d'hémiplégie gauche. Le diagnostic d'accident vasculaire cérébral (AVC) récent au territoire sylvien total droit a été confirmé par une tomodensitométrie (TDM) cérébrale. Le patient a été alors mis sous traitement antiagrégant plaquettaire type acide salicylique à la dose de 100 mg/j et une anticoagulation par héparine de bas poids moléculaire (HBPM) à dose préventive. Au cinquième jour de son hospitalisation, le patient a présenté brutalement un syndrome abdominal aigue. A l'examen, le patient était apyrétique, la tension artérielle était de 85/55 mmHg avec une fréquence cardiaque à 110 et une polypnée à 30 cycles/minute. La palpation abdominale a révélé une défense de l'hémi-abdomen droit et une douleur du reste de l'abdomen. Un bilan biologique fait en urgence a montré une baisse de l'hémoglobine de 13,2 à 11,7 g/dl, une numération plaquettaire normale, un taux de prothrombine (TP) à 70%, un temps de céphaline activée (TCA) correct. Par ailleurs, il n'y avait ni hyperleucocytose ni une élévation du « C-reactive protein (CRP) ».

**Figure 1:**
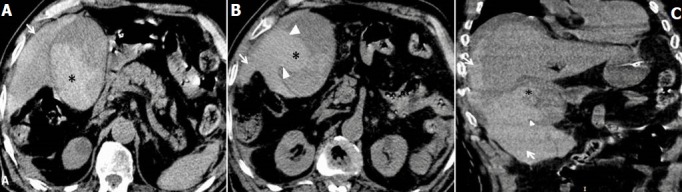
TDM abdominale sans injection de produit de contraste iodé (PDCI) en coupes axiales (A et B) et coronale (C); hématome intravésiculaire spontanément hyperdense (astérix noire) fusant en intrapéritonéal (flèche blanche) par une large rupture de la paroi de la vésicule biliaire (têtes de flèches blanches)

**Figure 2:**
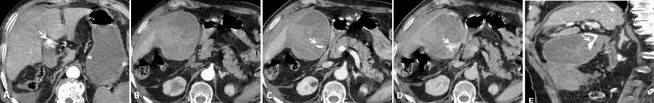
Angio-TDM abdominale sans injection de PDCI en coupes axiales respectivement aux temps artériel (A et B), portal (C) et tardif (D) et en coupe coronale (E) au temps portal; extravasation active du PDCI en intravésiculaire (flèche blanche) débutant dans le collet dès le temps artériel (A) avec propagation secondaire dans le corps et le fundus de la vésicule biliaire (B, C, D et E)

**Figure 3:**
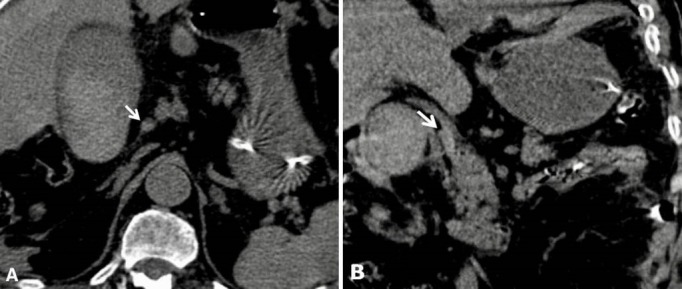
TDM abdominale sans injection de PDCI en coupes axiale (A) et coronale (B); hémobilie: contenu spontanément hyperdense de la voie biliaire principale (flèche blanche)

Le diagnostic évoqué, en premier lieu, était une ischémie mésentérique aigue. L'angio-TDM abdominale faite en urgence a montré une vésicule biliaire distendue, siège d'un contenu spontanément hyperdense (densité à 55-60 UH) fusant en intrapéritonéal et en péri hépatique à travers une large perforation de sa paroi latérale droite (Figure 1). Après injection intraveineuse de produit de contraste iodé (PDCI), on a noté dès le temps artériel, une extravasation active du PDCI, débutant au niveau du collet vésiculaire en regard de l'artère cystique et se propageant progressivement aux temps portal et tardif (Figure 2). L'étude de la voie biliaire principale notait également une hémobilie (Figure 3). Le diagnostic d'hémocholécyste compliqué d'une rupture de la vésicule biliaire et d'un hémopéritoine de grande abondance a été retenu. Le patient a été opéré en urgence. L'exploration per opératoire a montré la présence d'un hémopéritoine de grande abondance avec une vésicule biliaire partiellement décollé de son lit et perforée sur 2 cm au niveau de sa face antérieure. On a noté aussi que la présence d'un saignement actif venait de l'intérieur de la vésicule biliaire et à partir du lit vésiculaire décollé. Le geste a consisté en une cholécystectomie, une hémostase du lit vésiculaire et une évacuation de l'hémopéritoine. La vésicule biliaire était alithiasique. L'examen anatomopathologique a conclu à une cholécystite chronique modérée compliquée d'une poussée aiguë. L'évolution au cours des 4 premiers jours post opératoires a été marquée par une instabilité hémodynamique (alternance de pics hypertensifs et d'hypotension sévère) et une aggravation de l'état neurologique associant une altération de l'état de conscience et un passage en anisocorie droite. Une TDM cérébrale en urgence a montré une extension de l'AVC vers tout le territoire carotidien interne droit avec un engagement temporal interne homolatéral. Le patient est décédé à J4 post opératoire.

## Discussion

C'est une pathologie très rare, environ 65 cas d'hémocholécyste sous anticoagulants ont été rapportés dans la littérature [1-4]. La particularité de notre observation est la survenue de l'hémocholécyste sous une dose préventive de HBPM. Elle est plus fréquente en cas de cholécystite chronique et souvent secondaire à une effraction de l'artère cystique par l'irritation d'un calcul enclavé au niveau du collet vésiculaire ou à une ulcération par des calculs qui érodent la paroi vésiculaire [5]. L'hypocoagulabilité, induite par les anticoagulants et les antiagrégants plaquettaires, aggrave l'hémorragie intra vésiculaire ce qui peut entrainer une perforation de la vésicule biliaire avec passage du sang dans la cavité péritonéale constituant un hémopéritoine souvent de grande abondance. La zone de perforation habituelle se situe au niveau du fundus, moins vascularisée et plus sensible à l'hyperpression intra vésiculaire [6]. L'hémocholécyste reste asymptomatique dans la majorité des cas [1, 2]. Il peut se présenter comme un tableau de cholécystite aigue ou plus rarement dans un tableau d'état de choc hémorragique avec douleur abdominale. La TDM abdominale est l'examen clé pour le diagnostic positif. Il montre souvent une masse sous hépatique prenant la forme d'une vésicule biliaire dilatée, présentant un contenu hémorragique. Il faut chercher une perforation, un épanchement intra-péritonéal ou une compression d'un organe de voisinage. L'injection de PDC permet d'étudier la paroi vésiculaire et chercher un saignement actif visible sous la forme d'une extravasation de PDC.

## Conclusion

Malgré la rareté de la traduction clinique des hémorragies intra vésiculaires, l'hémocholécyste doit être évoqué chez tout patient sous anticoagulants et/ou antiagrégant plaquettaire présentant un tableau douloureux abdominal. La perforation de la vésicule biliaire responsable d'un hémopéritoine et d'un choc hémorragique est la complication la plus redoutée.

## Conflits d’intérêts

Les auteurs ne déclarent aucun conflit d'intérêts.
